# Benign mass-like lesion at the cholecystectomy bed: A case report of unusual postcholecystectomy imaging findings

**DOI:** 10.1016/j.radcr.2026.01.035

**Published:** 2026-02-13

**Authors:** Niloofar Ayoobi Yazdi, Mohammadreza Tahamtan, Shahriar Rahmani, Faeze Salahshour, Sajjad Alizadeh, Roberto Cannella

**Affiliations:** a Advanced Diagnostic and Interventional Radiology Research Center (ADIR), Tehran University of Medical Sciences, Tehran, Iran; b Department of Pathology, School of medicine, Tehran University of Medical Sciences, Tehran, Iran; c Department of Biomedicine, Neuroscience and Advanced Diagnostics (BiND), Section of Radiology, University of Palermo, Palermo, Italy

**Keywords:** Postcholecystectomy complications, Mass-like lesion, Imaging, Ultrasound, MRI, CT differential diagnosis

## Abstract

Cholecystectomy is associated with a variety of complications. Imaging plays a pivotal role in diagnosing these complications, but some imaging findings are poorly understood and unexplored. Here, we present a 59-year-old woman with a mass-like solid-cystic lesion at the cholecystectomy bed after 12 years of surgery. The lesion was hypoechoic in ultrasound, hypodense in CT scan, and had low T2 and intermediate T1 signal intensity and no enhancement in MRI, which was not consistent with the imaging characteristics of known complications. Histopathological evaluation revealed foreign body fragments and fibrin materials within the lesion. We conclude that radiologists should be familiar with these findings to avoid unnecessary interventions and misdiagnosis.

## Introduction

Cholecystectomy is one of the most frequently performed surgical procedures annually. It is associated with various postoperative complications [1]. The main indications for cholecystectomy are biliary colic, acute cholecystitis, chronic cholecystitis, polyps, and gallbladder masses [2]. After the widespread of laparoscopic cholecystectomy, the majority of procedures are done with this approach mainly due to its faster recovery and lower cost [3]. Although laparoscopic cholecystectomy is the preferred operative approach, it is associated with a higher incidence of major complications compared to open cholecystectomy. Notably, bile duct injury occurs and has an estimated incidence between 0.5% and 1.5% [4]. Given the widespread use of this procedure, the overall prevalence of complications remains significant [5].

Imaging plays a crucial role in the prompt and accurate diagnosis of complications following cholecystectomy. Modalities such as ultrasound, CT, and MRI are standard imaging methods for identifying postoperative complications such as biliary leaks, vascular injuries, biliary strictures, retained cholelithiasis [6]. The rapid and noninvasive imaging diagnosis of these complications can reduce patient morbidity and eliminate the need for unnecessary invasive procedures [[7], [8], [9]].

Using a hemostatic agent in the surgical procedure such as a sponge or Surgicel, which is oxidized regenerated cellulose, can also mimic pathological collections in the gallbladder fossa. Unlike nondegradable surgical sponges, which may lead to gossypibomas if inadvertently retained, these agents are often intentionally left in the surgical bed, particularly when postoperative oozing is anticipated, such as in patients undergoing major surgery or receiving anticoagulation therapy [10]. Although multiple types and brands of hemostatic agents exist, they share overlapping imaging appearances that can potentially mimic a variety of abdominal abnormalities [11]. They appear as focal air collections with heterogenous density on CT. The absence of radiopaque material makes it difficult to differentiate from the pathological collection. On MRI, Surgicel typically exhibits a low T2-signal, in contrast to most pathological collections, which display a high T2-signal. Additionally, reviewing the surgical report can aid in differentiating between hemostatic agents and pathological collections [12,13].

This article presents a postcholecystectomy patient with mass-like lesion at the surgical bed that are not compatible with previously reported complications, such as biloma or fluid collections.

## Case presentation

A 59-year-old woman was referred for assessment of a lesion identified on a CT scan during an evaluation for right upper quadrant (RUQ) pain. The patient presented with abdominal pain, which prompted postoperative imaging evaluation. The pain was not directly related to the lesion identified at the cholecystectomy bed, as imaging did not demonstrate features of an acute postoperative complication such as abscess formation, active bleeding, biliary leak, or bowel injury. Laboratory investigations were unremarkable, with no evidence of infection or inflammatory response. The patient was managed conservatively with symptomatic treatment, and the abdominal pain resolved over time without the need for further intervention. On follow-up imaging, the lesion remained stable in size and imaging characteristics, and no additional corroborative clinical or radiologic findings were identified to suggest a pathologic cause for the initial pain.

**Fig. 1:**
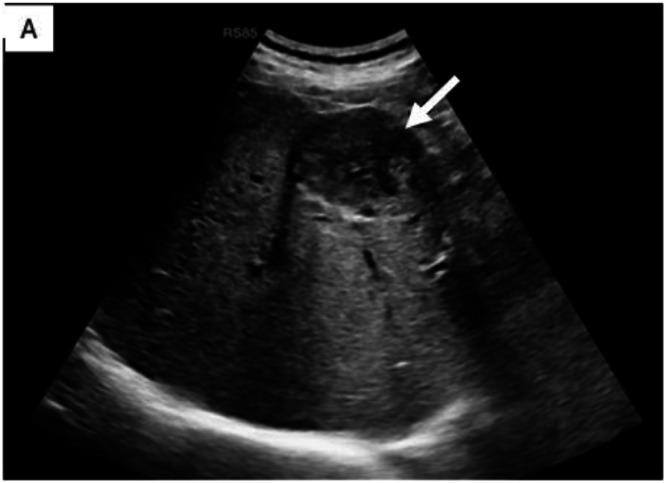
Ultrasound image shows a solid-cystic echoic lesion with posterior acoustic enhancement in cholecystectomy bed (white arrow) (A).

**Fig. 2:**
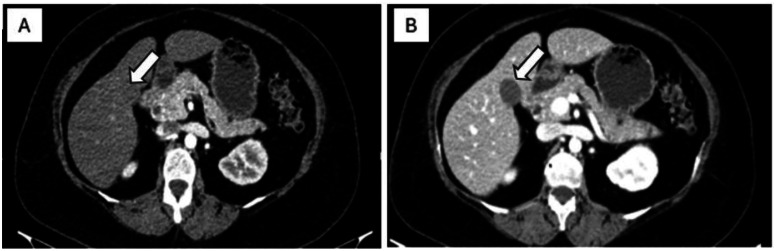
Abdominopelvic contrast-enhanced CT scan images of the patient with postcholecystectomy lesion. Postcontrast abdominal CT scan image shows non enhancing lesion with low density in the bed of cholecystectomy (white arrows). A hypodense lesion in postcontrast arterial (A), and venous (B)phase CT scan image.

**Fig. 3:**
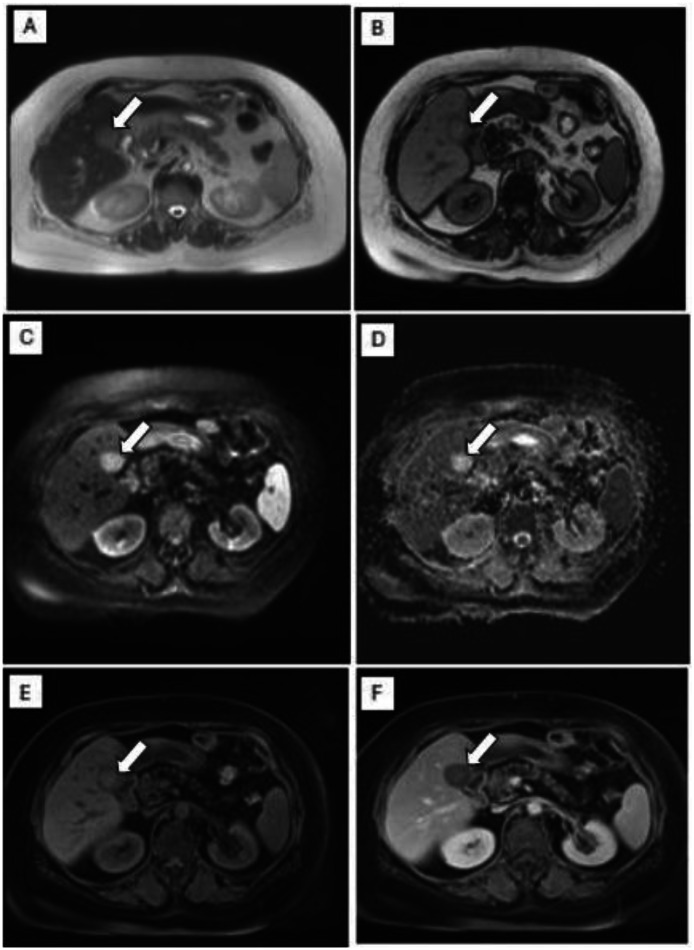
MR images of a patient with a postcholecystectomy lesion located in the cholecystectomy bed, indicated by the white arrow on all images. The lesion (white arrow) demonstrates intermediate signal intensity on T2-weighted SS-FSE images (A) and low-to-intermediate signal intensity on T1-weighted opposed-phase images (B). The lesion marked by the white arrow appears hyperintense on both DWI (C) and ADC maps (D), consistent with the absence of diffusion restriction. On noncontrast T1-weighted imaging (E), the lesion identified by the white arrow shows no intrinsic hyperintensity, and no enhancement is observed on postcontrast T1-weighted images (F).

On US, a well-circumscribed relatively round hypoechoic mixed solid cystic lesion at the bed of the surgery was seen. On Color Doppler examination the lesions didn’t show any vascularity (Fig. 1). CT demonstrated hypodense round mass-like lesions at the bed of the surgery. No evidence of metal artifact, air or fat is noted at surgical bed. There was no evidence of perilesional fat stranding or invasion to the adjacent structures. No contrast enhancement was detected (Fig. 2). MRI revealed an intermediate T2 and low to intermediate T1 signal intensity. No diffusion restriction on the DWI was observed nor signal drops on in phase-out phase. No enhancement was detected on the postcontrast T1-weighted imaging (Fig. 3). Follow-up imaging was assessed and was compared with previous studies according to size, shape, and enhancement pattern showing lack of significant change in size or morphology or new enhancement over a follow-up period. Imaging features of the presented cases were summarized in Table 1.

**Table 1:** Summary of imaging findings of the present case across ultrasound, CT, and MRI modalities.

Imaging features	Present case
ultrasound	Solid–cystic lesion with predominantly hypoechoic echotexture
Color doppler	Absence of internal vascularity
CT scan	Hypodense mass like lesion
enhancement	No enhancement after contrast administration
MRI signal characteristics	Low signal intensity on T1-weighted images and intermediate signal intensity on T2-weighted images
Diffusion restriction	No diffusion restriction
Additional features	Stable appearance on follow-up imaging

**Fig. 4:**
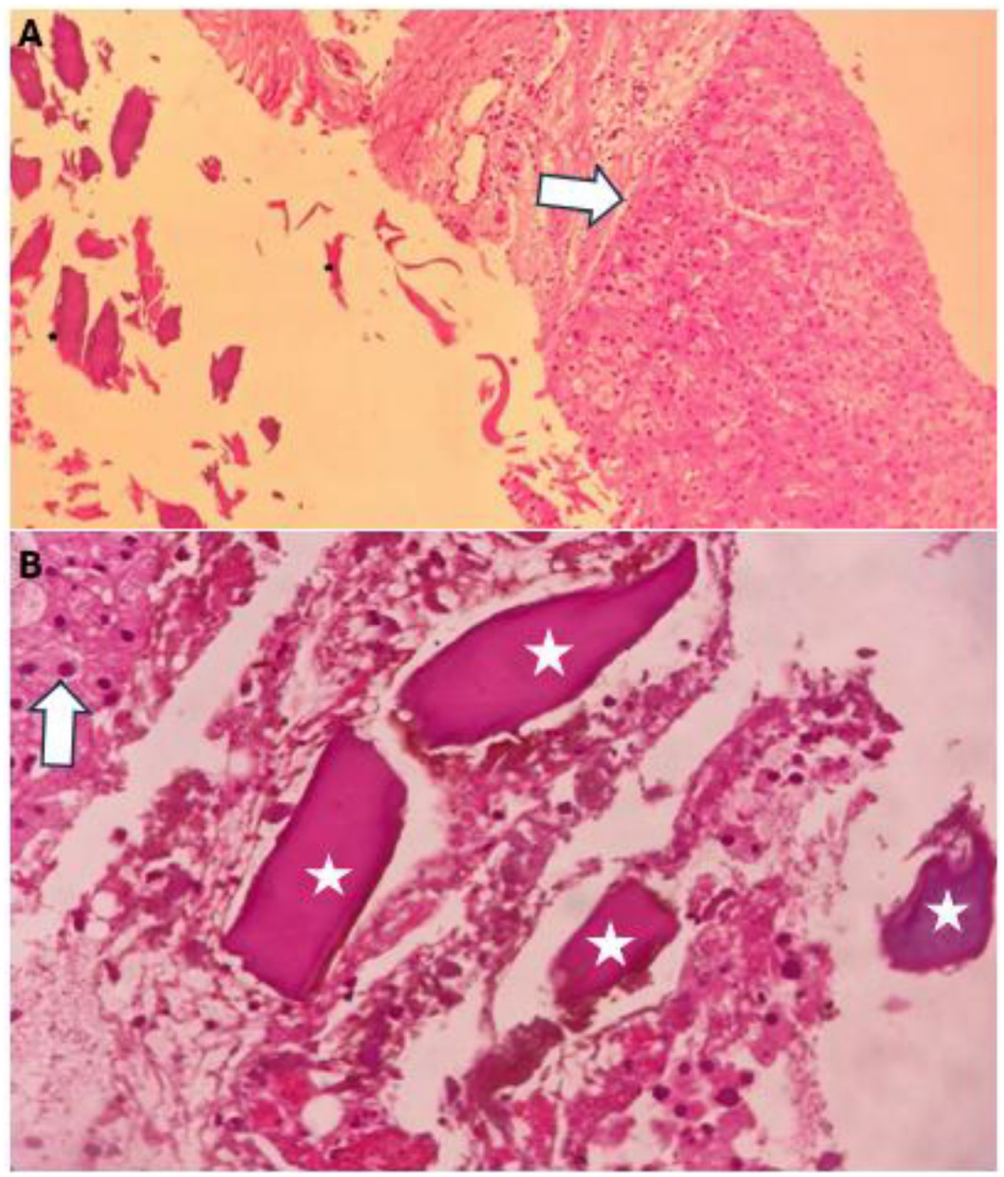
(A) Acellular foreign body and fibrin materials (*) near hepatocytes and liver capsule (white arrow) without significant inflammatory response, (B) Closer view (400x) of the homogenous foreign body materials (*) near hepatocytes (white arrow).

The patient underwent a core needle biopsy of the lesion due to the referring clinician's suspicion of malignancy. The histopathologic evaluation of the biopsy revealed no malignant cells but identified fragments of foreign body and fibrin materials within the lesion (Fig. 4).

## Discussion

The use of surgical hemostats and sealants during operations has grown substantially in recent years. This rise is primarily driven by the shift toward minimally invasive and percutaneous procedures [10]. Their postprocedural imaging appearances have been described in the literature following various abdominal and pelvic surgeries, most commonly in the urologic context, particularly after partial nephrectomy. However, to our knowledge, no prior reports have described these findings following cholecystectomy; therefore, we present this case to address this gap.

Topical hemostatic agents are newer adjuncts to conventional techniques and may be used when standard methods are insufficient or impractical, either due to the severity of bleeding or the surgical location. These agents are particularly effective for controlling hemorrhage from complex or difficult-to-access sites, especially during minimally invasive procedures with a limited field of view [14]. Hemostatic agents comprise various materials used to control bleeding, including microfibrillar collagen products (with or without thrombin), gelatin-based matrices, oxidized regenerated cellulose, and polysaccharide-based agents. After placement, it absorbs blood and fluid, forming a gelatinous mass that can mimic soft tissue, fluid collections, or abscesses on imaging [10].

Cholecystectomy is among the most commonly performed surgical procedures. Despite its overall safety, the high volume of operations leads to a significant incidence of postcholecystectomy complications [12]. Cholecystectomy is generally regarded as a low-risk procedure, and its postoperative complications differ from those of more complex surgeries, often being less severe. However, prompt recognition of these complications by radiologists can benefit patients by reducing costs and avoiding unnecessary invasive interventions [15]. This study aimed to highlight an unexamined multimodality radiological finding observed in a postcholecystectomy patient, which should not be misinterpreted as other similar complications, thereby avoiding unnecessary invasive procedures.

We identified a mass-like lesion at the cholecystectomy bed that exhibited no enhancement, and no vascularity on color Doppler. In patients with a history of cholecystectomy, the most common differential diagnoses for such findings would be biloma, hematoma, abscess formation, and Surgicel insertion during surgery. However, the findings were not consistent with biloma, hematoma, nor abscess formation. Additionally, the presence of mass-like lesions in the cholecystectomy bed could mimic gallbladder malignancy. Incidental gallbladder cancer diagnosed after laparoscopic cholecystectomy is not rare [16]. Imaging findings of gallbladder cancer may include irregular borders, an invasive mass at gallbladder fossa, intrahepatic biliary dilatation, invasion of adjacent structures, lymphadenopathy and peripheral enhancement on postcontrast imaging. However, none of these features were observed in our study [17].

Bilomas typically appear as thin-walled cystic lesions on ultrasound and CT, showing no color Doppler flow or contrast enhancement. MRI is particularly valuable for differentiating bilomas from other postoperative entities, such as subacute hematomas. On MRI, bilomas are usually hyperintense on T2-weighted images and may occasionally show layering with high T1 and low T2 signal due to concentrated bile [18]. In our case, however, the lesion demonstrated lower T2 signal intensity than expected for a biloma. Hematomas represent another postoperative consideration and generally resolve spontaneously within months. Because the lesions in our case appeared several years after surgery, an acute or subacute hematoma was unlikely. Although rare cases of chronic organized hematomas that fail to resorb have been reported, these typically show low signal intensity on both T1- and T2-weighted images, sometimes with heterogeneous central T2 hyperintensity [19]. The MRI features observed in our case were not consistent with those described for chronic hematomas.

Hemostatic agent like surgicel is often used during surgery to achieve hemostasis. Although Surgicel can be quickly resorbed, it may persist for 4-8 weeks in the body and even years after the initial surgery [18]. Residual Surgicel in the body may be mistaken for complications such as hematoma, abscess, or tumor recurrence during postsurgical paraclinical examinations. T2-weighted MRI images are particularly useful in accurately diagnosing Surgicel, as it typically presents with a low T2 signal [19]. On US, surgicel typically appears as a complex, mass-like structure with heterogeneous hypoechoic and hyperechoic areas. Its most common appearance on CT includes focal collections of gas within mixed-attenuation masses, which is incompatible with our observations [13]. Although the insertion of Surgicel was not documented in our case, the observed lesions exhibited low to intermediate T2 signal with no enhancement, leading us to hypothesize that the lesions could be residual Surgicel. This hypothesis is further supported by the presence of foreign body material and fibrin in histopathological examination.

Surgical site abscesses may mimic Surgicel on imaging, but MRI helps differentiate them, as abscesses typically show high T2 signal intensity, whereas Surgicel appears hypointense on T2-weighted images [19,20]. In our case, abscess formation was excluded based on the low T2 signal and the absence of clinical signs of infection. Gallbladder perforation with spillage of gallstones (“dropped stones”) is a known complication of laparoscopic cholecystectomy, but its imaging features differ from our findings, with stones appearing hyperechoic on ultrasound and typically hypointense on both T1- and T2-weighted MRI sequences without enhancement [8,21].

Although several suggestive imaging features have been outlined, the most critical factor for the radiologist is awareness of the patient’s surgical history and the potential use of hemostatic agents. In one study, diagnostic accuracy for identifying a cellulose-based agent increased from 11% to 83% once the radiologist was informed of its use during recent surgery [10]. The presence of sutures or surgical clips in association with these lesions should further alert the radiologist to consider these possibilities [10]. Finally, this entity should be distinguished from an accidentally retained foreign body after surgery, known as a gossypiboma. Gossypibomas typically demonstrate a spongiform appearance with internal gas bubbles on CT imaging; however, the most informative distinguishing feature is their lack of resolution over time. Therefore, careful consideration of the patient’s surgical history is essential to avoid misdiagnosis [22].

Across all imaging modalities, our patient demonstrated a mass-like lesion at the cholecystectomy bed, which was suggestive of residual hemostatic agents in gallbladder fossa. We hypothesize that these lesions represent postsurgical changes, likely due to Surgicel insertion in cholecystectomy patients, supported by histopathological confirmation. Importantly, this lesion should not be misinterpreted as serious postoperative complications like bilomas or infectious collections. Moreover, the absence of malignant imaging features—such as invasion into surrounding structures, postcontrast enhancement, or other aggressive findings—rules out malignancy. As such, invasive procedures should be avoided due to the likely benign nature of these lesions. It should be considered that the absence of detailed surgical histories, particularly regarding Surgicel insertion, limited our ability to fully correlate the imaging findings with specific intraoperative or postoperative factors. A comparison between residual hemostatic agents and other differential diagnoses of postcholecystectomy bed lesions is summarized in Table 2.

**Table 2:** Comparative imaging characteristics of postcholecystectomy bed lesions.

	retained hemostatic agents	bilomas	Chronic hematomas	abscesses	malignancies
**Ultrasound**	Heterogenous mass-like lesion	Cystic lesion	Heterogeneous appearance	Fluid collection with internal echoes	Echogenic to isoechoic mass
**Color doppler**	No internal vascularity	No internal vascularity	No internal vascularity	Peripheral Wall vascularity	internal vascularity
**CT scan**	Heterogeneous mass-like lesion with gas foci	Hypo dense cystic lesion	Heterogeneous lesion with iso- to hyperdense components	Hypodense lesion	Hypodense or isodense lesion
**Contrast enhancement**	No contrast enhancement	No contrast enhancement	No contrast enhancement	Rim enhancement	Peripheral or heterogeneous enhancement
**MRI signal**	variable T1 Low T2	Low T1 High T2	Low T1 Low to heterogeneously high T2	Low T1 Hight T2	Hypo-to-isointense T1 iso-to-hyperintense T2
**Diffusion restriction**	Absent	Absent	variable	Present	May be present
Additional features					Irregular border, associated lymphadenopathy, and invasion to adherent structures

In conclusion, accurate radiological interpretation is essential in post cholecystectomy cases to avoid unnecessary invasive interventions. Our study highlights the importance of differentiating between hemostatic agents and other potential complications such as bilomas, hematomas, and abscesses. The lesion observed in this case exhibited characteristics consistent with residual surgical foreign material, supported by histopathological evidence in core needle biopsy. These findings emphasize the benign nature of such lesions, which remain stable in size and shape over time. Recognizing these imaging characteristics can prevent misdiagnosis, avoid unnecessary procedures, and contribute to better patient outcomes by reducing costs and preventing unnecessary interventions.

## Patient consent

Written, informed consent was obtained from the patient for publication of this case.
